# Epidemiology and disease burden of tuberous sclerosis complex in France: A population‐based study based on national health insurance data

**DOI:** 10.1002/epi4.12636

**Published:** 2022-08-27

**Authors:** Francis Fagnani, Caroline Laurendeau, Marie de Zelicourt, Jade Marshall

**Affiliations:** ^1^ Cemka Bourg‐la‐Reine France; ^2^ Jazz Pharmaceuticals, Inc. London UK

**Keywords:** healthcare cost, healthcare resource utilization, prevalence, refractory epilepsy, TSC

## Abstract

**Objective:**

Tuberous sclerosis complex (TSC) is a rare multisystem disorder, often associated with epilepsy. This retrospective study aimed to identify patients with TSC, including those with epilepsy, from a French healthcare claims database, and to report incidence, prevalence, and healthcare costs and resource utilization.

**Methods:**

The anonymized French health insurance database (SNDS) covers almost the entire French population. Patients with TSC were identified as having ≥1 International Classification of Diseases, Tenth Revision (ICD‐10) diagnosis code Q85.1 or a long‐term disease (LTD) registration over the inclusion period (2006–2017). Patients with an ICD‐10 epilepsy code or who were dispensed ≥1 antiseizure medication (ASM) in the same year or after their TSC diagnosis were identified as having TSC with epilepsy. Newly diagnosed patients over the inclusion period constituted the incident cohort. Healthcare costs (patients with recorded costs only), healthcare resource use, and ASM dispensation are reported for patients with 2018 data.

**Results:**

In 2018, 3139 prevalent patients with TSC were identified (crude prevalence, 4.69 per 100 000); the incident cohort comprised 2988 patients (crude incidence, 0.44 per 100 000). Among patients with TSC, 67% (2101/3139) had epilepsy (mean [standard deviation, SD] age: 28.8 [18.8] years; male: 48%). Among patients with epilepsy, total mean (SD) annual healthcare costs were €11 413 (27 620) per capita (outpatient, 63%; inpatient, 37%), 46% were hospitalized during 2018 (mean [SD]: 1.8 [10.9] acute care visits per patient), and 65% visited a hospital specialist. Among patients with epilepsy, medication (mean [SD]: €4518 [12 102] per capita) was the greatest contributor (63%) to outpatient costs, and in 2018, 74% were dispensed ≥1 different ASM and 9% were dispensed ≥4 ASMs.

**Significance:**

TSC with epilepsy was associated with substantial healthcare costs and resource utilization, particularly outpatient and medication costs. Many patients with TSC with epilepsy were prescribed multiple ASMs, suggesting refractory epilepsy.

**Key Points**

- This is the largest retrospective population‐based study in tuberous sclerosis complex (TSC) in a French population, to date.
- TSC was associated with considerable healthcare costs and utilization, particularly in patients with epilepsy.
- A large proportion of outpatient costs in patients with TSC with epilepsy (63%) were attributed to medication.
- Many patients with epilepsy were dispensed multiple antiseizure medications, suggesting a high rate of refractory epilepsy.
- Rates of intellectual disability were higher in patients with TSC with epilepsy compared with those without epilepsy.

## 1 INTRODUCTION

Tuberous sclerosis complex (TSC) is a rare multisystem genetic disorder characterized by benign tumors in multiple organs, including the skin, brain, kidneys, lungs, and heart, but disease presentation and severity vary widely among individuals.^1^, ^2^ A genetic diagnosis of TSC is based on the identification of a heterozygous pathogenic mutation in either the *TSC1* or *TSC2* gene.^2^ However, 10%–25% of patients with TSC have no identifiable mutation; thus, clinical diagnostic criteria, such as dermatological or neurological features of TSC, may also be used to confirm the diagnosis.^2^

Epilepsy is a common neurological symptom associated with TSC that has previously been estimated to be present in between 63% and 93% of patients with TSC.^3^, ^4^, ^5^, ^6^, ^7^, ^8^, ^9^, ^10^, ^11^ Many of these patients develop refractory epilepsy, with previous estimates of refractory epilepsy in patients with TSC with epilepsy of between 63% and 78%.^5^, ^12^

Previously published data on healthcare resource use and healthcare costs in patients with TSC have consisted of retrospective cohort studies using data sourced from health insurance or national databases,^13^, ^14^, ^15^ as well as cross‐sectional^16^ or web‐based surveys,^17^ which have been conducted at a national or multicenter level. The evidence suggests that TSC is associated with high burden of illness, in terms of healthcare resource utilization and associated costs, although the individual healthcare needs of patients with TSC can vary greatly depending on the number and severity of disease manifestations.^13^, ^14^, ^15^, ^16^, ^17^ In particular, epilepsy has been associated with greater healthcare costs in patients with TSC,^15^ although few studies have specifically assessed the burden of epilepsy on healthcare resource use and healthcare costs,^9^, ^10^, ^18^, ^19^, ^20^ and to the best of our knowledge no studies have been conducted in France.

The aim of this retrospective French healthcare claims database analysis was to examine prevalence, incidence, healthcare costs and resource utilization, medication use, comorbidities, and survival in patients with TSC, with a focus on those with epilepsy.

## 2 METHODS

### 2.1 Data source

The Système National des Données de Santé (SNDS) is composed of the French national healthcare claims database, the national hospital discharge database, and the national death registry.^21^ The SNDS includes information on all items of reimbursed healthcare utilization, including hospital admissions, outpatient visits, and all medications dispensed by community pharmacists for almost the entire population in France (98.8%).^21^ Several previous studies have used SNDS data for publications on incidence of disease, adverse event assessment of drugs, monitoring of national health insurance expenditure, and real‐life use of drugs. Permission to access data is granted depending on what type of data are requested and researcher's status.^22^

In the French healthcare system, long‐term disease (LTD) status means that a patient has a severe chronic disease requiring prolonged and costly therapy, enabling them to receive full insurance coverage for all medical expenses related to this condition. LTD registration must be requested by the patient's treating physician and validated by a health insurance scheme physician.^21^ In total, 30 groups of severe chronic diseases are covered by LTD status in France, although in practice, almost all serious chronic conditions are covered by the LTD arrangements.^23^ The International Classification of Diseases, Tenth Revision (ICD‐10) code under which the LTD is registered and the date of first registration are recorded in the SNDS.^21^

### 2.2 Ethical approval

This study was conducted in accordance with relevant international and French regulatory requirements. Patient data in the database were anonymized using an irreversible double encryption. Since this was a retrospective study of an anonymized database and had no influence on patient care, ethics committee approval was not required. Use of the SNDS database for this type of study is regulated by the French national data protection agency (Commission Nationale de l'Informatique et des Libertés), and the project formally approved under the Decision DR 2019–324.

### 2.3 Patient identification and inclusion periods

**FIGURE 1:**
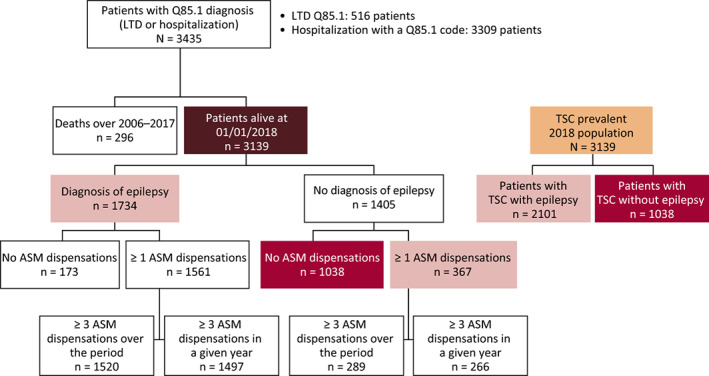
Patient identification algorithm for TSC and TSC with epilepsy (prevalent population). Abbreviations: ASM, antiseizure medication; LTD, long‐term disease

To identify the TSC population, data were assessed over a 13‐year period from January 1, 2006 to December 31, 2018. Patients with TSC were identified during this period if they had been recorded as having at least one TSC diagnosis (ICD‐10 code: Q85.1) during a hospitalization, or if they had an LTD registration for TSC (Figure 1). The prevalent 2018 population included patients with prior evidence of TSC who were alive on January 1, 2018.

As part of a sensitivity analysis for the identification of the prevalent population, another set of patients with probable TSC were identified as those who had the same inclusion criteria as above but adding an LTD registration under the ICD‐10 code Q85* (Phakomatoses, not elsewhere classified). In this population, we secondarily excluded patients who were hospitalized with a diagnosis recorded as ICD‐10 code Q85.0 (Neurofibromatosis), Q85.8 (Other neurofibromatosis, not elsewhere classified), or Q85.9 (Phakomatosis, unspecified). This wider selection process was performed to obtain an upper range estimate of the prevalent 2018 TSC cases.

The incident population was defined as all patients who had at least 2 years of documentation history prior to their index date (T0, date of first TSC diagnosis found in the database) without TSC diagnosis. To ensure at least 1 year of follow‐up for each patient, the inclusion period for the incident TSC population was from January 1, 2008 to December 31, 2017. The end of the follow‐up period was defined by one of the following events: last health record of the patient (eg, the last recorded consultation, prescription, or medical procedure) in the database before a 6‐month period without any reimbursed care, death of the patient, or the end of the study period (December 31, 2018).

The identified TSC patient group was divided into two subgroups: TSC with epilepsy and TSC without epilepsy. Patients with epilepsy were identified as having an additional diagnosis of epilepsy (ICD‐10 code G40* or G41*) or at least one dispensed antiseizure medication (ASM; Anatomical Therapeutic Chemical Classification System code N03A), commonly referred to as antiepileptic drugs, in the same year as TSC diagnosis or later.

### 2.4 Outcomes

Outcomes included prevalence, incidence, demographic characteristics (age and sex), all‐cause annual healthcare costs (measured in Euros), healthcare resource utilization (hospitalizations [inpatient] and ambulatory medical care [outpatient] visits), and dispensed medication. Dispensed medications included ASMs, commonly referred to as antiepileptic drugs, which were identified by their Anatomical Therapeutic Chemical Classification System (code N03A), and everolimus (Votubia®; licensed in Europe for treatment of subependymal giant cell astrocytomas associated with TSC, renal angiomyolipomas associated with TSC, and patients with treatment‐refractory partial‐onset seizures associated with TSC),^24^, ^25^ for which formulations were identified by their corresponding Club Inter Pharmaceutique codes. Outcomes also included comorbidities, identified according to LTD registration ICD‐10.1 codes, survival, evaluated using a Kaplan–Meier model from index date, and mortality.

Prevalence, demographic characteristics, healthcare costs, healthcare resource utilization, and comorbidities are reported using the prevalent population for the most recent documented year (2018). Incidence, reported for all patients with TSC, and survival and mortality, reported for patients with TSC with and without epilepsy, are based on the incident population. Dispensed medications are reported for the prevalent and incident populations.

### 2.5 Economic analyses

The SNDS database provides all healthcare consumptions for each patient, including outpatient (ambulatory) and inpatient (hospitalization) care. Inpatient care is documented for acute care facilities, rehabilitation, home care, and psychiatric hospitals. Some specialized institutions involved in the care of patients with severe TSC may not be captured in this analysis.

SNDS provides outpatient expenses data for amounts effectively reimbursed and out of pocket expenses. In both cases, only direct costs were considered.

For inpatient data, cost estimations followed the National Authority for Health (Haute Autorité de Santé) guidelines,^26^ sourcing data from the national hospital costs study database (ENCC, Étude nationale de coûts méthodologie commune)^27^ to represent average costs, which account for variations between facilities (eg, public and private hospitals) and are based on accounting data.

### 2.6 Statistical analyses

Data are summarized descriptively, with mean and standard deviation presented for continuous variables, and frequency, including numbers and percentages, presented for categorical variables. Kaplan–Meier survival analyses and the log‐rank test were used to evaluate survival and perform comparisons between subgroups.

All statistical analyses were performed using SAS® software, version 9.4.0 (North Carolina, USA).

## 3 RESULTS

### 3.1 Prevalence and incidence

The prevalent population (2018) comprised a total of 3139 patients with TSC (Figure 1). Most patients (96%) were identified on the basis of hospitalization with the ICD‐10 code Q85.1, while fewer patients (15%) had LTD status (Q85.1) for TSC. The corresponding crude prevalence rate was 4.69 per 100 000. From this population, 67% (2101/3139) had TSC with epilepsy. Through the wider selection process (LTD registration code Q85* after exclusion of patients with a history of hospitalization mentioning the ICD‐10 codes Q85.0, Q85.8, or Q85.9), a total of 3471 patients were identified, with a corresponding prevalence of 5.19 per 100 000. Of these patients, 70% (2443/3471) had TSC with epilepsy.

The incident population comprised 2988 patients, corresponding to a mean annual incidence of 299 cases per year, that is, a crude mean annual incidence rate of 0.44 per 100 000.

### 3.2 Patient demographics

In the prevalent population, the mean (standard deviation [SD]) age for the overall TSC population in 2018 was 30.4 (19.6) years, and 46% of patients were male. These characteristics were similar for patients with epilepsy (mean [SD] age: 28.8 [18.8] years; male: 48%) and without epilepsy (mean [SD] age: 33.6 [20.7] years; male: 41%). The distribution of patients by age group and epilepsy status is presented in Figure S1.

### 3.3 Annual per capita healthcare costs

#### 3.3.1 All patients

**FIGURE 2:**
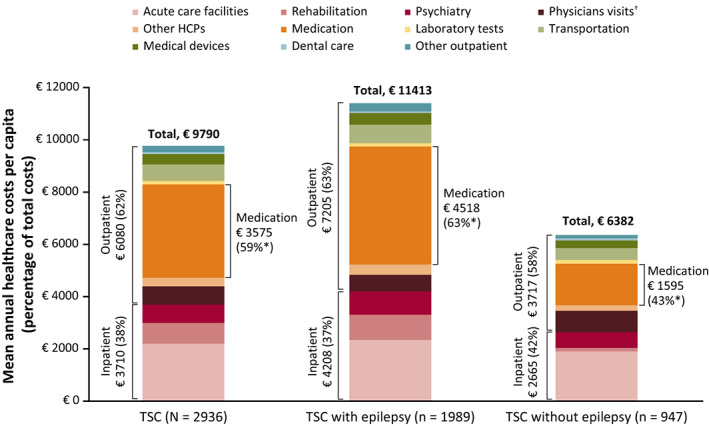
Annual healthcare costs for patients with TSC in 2018 (prevalent population, patients with recorded costs). *Percentage of outpatient care costs; ^†^Including hospital. Abbreviations: ASM, antiseizure medication; HCP, healthcare professional; TSC, tuberous sclerosis complex. Further details of costs are provided in Table S1.

Annual healthcare costs for the prevalent population during 2018 are reported in Figure 2 and Table S1. In patients with TSC, total mean (SD) annual healthcare costs were €9790 (24 531), of which almost two‐thirds comprised outpatient care. The largest contributor to outpatient care was medication costs (€3575 [10 589]). Meanwhile, acute care facilities contributed most to inpatient care costs (€2205 [6752]).

#### 3.3.2 Patients with epilepsy

Patients with epilepsy had higher annual healthcare costs (both inpatient and outpatient costs) compared with patients without epilepsy (Figure 2). The most notable difference was seen in medication costs, which were 2.8 times higher in patients with epilepsy than those without. Most of the medication costs for patients with epilepsy were attributed to everolimus (70%), while ASMs made up only a small proportion of the cost of medication (12%). All components of the inpatient costs were greater in patients with epilepsy, although acute care facilities accounted for a greater proportion of inpatient care costs for patients without epilepsy.

### 3.4 Healthcare resource utilization

#### 3.4.1 All patients

**FIGURE 3:**
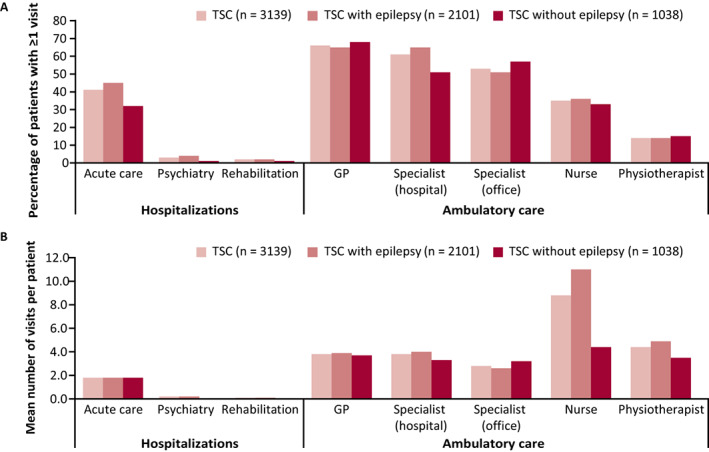
Annual hospitalizations and ambulatory medical care visits for patients with TSC in 2018. (A) Percentage of patients with ≥1 visit during 2018 and (B) mean number of visits in 2018 (prevalent population, all patients). Abbreviations: GP, general practitioner; TSC, tuberous sclerosis complex

Annual medical care visits and hospitalizations in the prevalent population during 2018 are reported in Figure 3A,B. In the overall TSC population, 41% of patients were hospitalized at least once (mean [SD] number of hospitalizations: 1.8 [11.7]), with most hospitalizations in acute care facilities. The majority of patients visited a general practitioner (66%, mean [SD]: 3.8 [5.4] visits) and/or hospital specialist (61%, mean [SD]: 3.8 [12.6] visits).

#### 3.4.2 Patients with epilepsy

A greater proportion of patients with epilepsy than without epilepsy were hospitalized (46% vs 32%) or visited a hospital specialist (65% vs 51%) at least once in 2018. The mean (SD) number of acute care hospitalizations was similar between patients with and without epilepsy (1.8 [10.9] vs 1.8 [13.0] per patient), while the number of hospital specialist visits was higher in patients with epilepsy (4.0 [12.8] vs 3.3 [12.2] per patient). Further to this, although a similar proportion of patients in each group visited a nurse (36% vs 33%) or a physiotherapist (14% vs 15%), patients with epilepsy had a higher mean (SD) number of visits in 2018 (nurse: 11.0 [51.6] vs 4.4 [23.9] per patient; physiotherapist: 4.9 [18.6] vs 3.5 (13.7) per patient). This higher frequency of visits in patients with TSC with epilepsy compared with those without epilepsy is particularly notable when the mean (SD) number of visits in 2018 is considered per consumer, that is, those who did require these services (nurse: 30.5 [82.2] vs 13.3 [40.2] per consumer; physiotherapist: 35.0 [37.9] vs 24.4 [28.2] per consumer).

### 3.5 Medication

**FIGURE 4:**
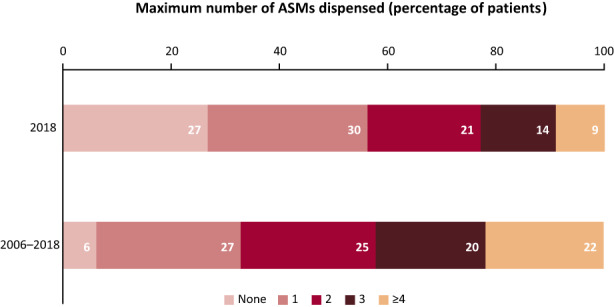
Dispensed ASMs for patients with TSC with epilepsy during 2018 (prevalent population) and between 2006 and 2018 (incident population). Abbreviations: ASM, antiseizure medication; TSC, tuberous sclerosis complex

Among patients with TSC with epilepsy, 74% of patients in the prevalent population were dispensed ≥1 ASM during 2018 and 94% were dispensed ≥1 ASM between 2006 and 2018 in the incident population (Figure 4). The most frequently dispensed ASMs in 2018 (percentage of patients receiving ≥1/≥3 dispensations) were carbamazepine (25%/23%), valproate (21%/19%), lamotrigine (18%/16%), vigabatrin (15%/14%), clobazam (15%/11%), and levetiracetam (13%/11%).

In 2018, everolimus was dispensed to 271/3139 (9%) of patients with TSC, including 11% of those with epilepsy and 4% of those without epilepsy.

### 3.6 Comorbidities

**FIGURE 5:**
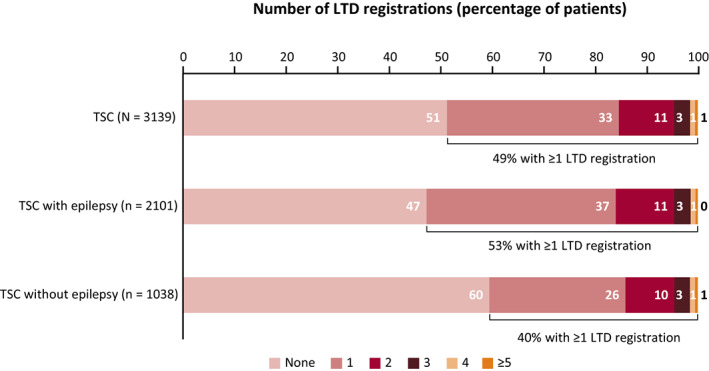
Number of comorbidities according to LTD registration code in patients with TSC in 2018 (prevalent population, all patients). Abbreviations: LTD, long‐term disease; TSC, tuberous sclerosis complex

In the prevalent population, 49% of patients with TSC had ≥1 comorbidity (Figure 5). The most common comorbidity was intellectual disability (15%) (Table 1), which was four‐fold more common in patients with epilepsy than without epilepsy (20% vs 5%).

**TABLE 1:** Types of comorbidities according to LTD registration code in patients with TSC in 2018 (prevalent population, all patients)

Diagnosis	ICD‐10 code	TSC (N = 3139) n (%)	TSC with epilepsy (n = 2101) n (%)	TSC without epilepsy (n = 1038) n (%)
Intellectual disability	F70–79	477 (15)	423 (20)	54 (5)
Other mental and behavioral disorders	F03–60	172 (6)	122 (6)	50 (5)
Other diseases of the nervous system	G00–71^a^	109 (4)	89 (4)	20 (2)
Congenital malformations, deformations and chromosomal abnormalities	Q0201–99^b^	99 (3)	56 (3)	43 (4)
Disorders of psychological development	F800–84	97 (3)	75 (4)	22 (2)
Metabolic disorders	E70–89	44 (1)	36 (2)	8 (1)
Cerebral palsy and other paralytic syndromes	G80–93	42 (1)	35 (2)	7 (1)
Status epilepticus	G41	8 (0.3)	8 (0.4)	0 (0)
Other		796 (25)	513 (24)	283 (27)

Abbreviations: ICD‐10, International Classification of Diseases, 10th version; LTD, long‐term disease; TSC, tuberous sclerosis complex.

^a^ Excluding G40–G41.

^b^ Excluding Q85.

### 3.7 Survival

Survival rates at 5 years post TSC diagnosis, based on analyses of the incident population, did not differ between patients with TSC with epilepsy (93%; n = 1864) and patients with TSC without epilepsy (94%; n = 996). Mean (SD) age at death was lower for patients with epilepsy (50.6 [18.4] years) than those without epilepsy (58.2 [21.7] years).

## 4 DISCUSSION

In this retrospective study, we used real‐world data from the French healthcare system, using the SNDS database, to determine prevalence, incidence, healthcare costs, healthcare resource utilization including medication use, comorbidities, and survival in patients with TSC in France. SNDS covers healthcare claims from 98.8% of the French population and around 600 million patient years of data.^21^ As such, this study adds valuable data about the epidemiology and disease burden of TSC to the existing literature; it should however be noted, when considering these results in the context of previous literature, that methodologies and outcome measures differ among studies.

The crude prevalence of TSC observed in this study was 4.69 per 100 000, which is consistent with that of previous prevalence estimates of between 3.8 and 8.8 per 100 000.^4^, ^10^, ^28^, ^29^, ^30^ The upper range estimate of TSC prevalence, determined here by a registration code of Q85 (excluding patients with a history of Q85.0, Q85.8, or Q85.9) instead of Q85.1, estimated a higher prevalence of 5.19 per 100 000. However, this estimate encompasses the wider LTD code for unspecified neurofibromatosis, and it is likely not all patients identified had TSC; as such, the study population was based on those with a more certain diagnosis.

This study found that around two‐thirds of patients (67%) with TSC had epilepsy, which is consistent with prevalence reported in previous studies (63–93%), although this is toward the lower range of previous estimates.^3^, ^4^, ^5^, ^6^, ^7^, ^8^, ^9^, ^10^, ^11^ A study using German health insurance data reported a low (36%) prevalence of epilepsy in patients with TSC,^14^ which the authors suggested might be explained by detection of more asymptomatic and mild cases of TSC due to increased family member screening. Additionally, prevalence may be overestimated in studies conducted in specialist TSC or epilepsy centers. Furthermore, when the upper range estimate was considered, the proportion of patients with TSC with epilepsy was slightly higher at 70%. Comorbidities other than epilepsy identified through LTD registrations, including intellectual disability and behavioral disorders, are also evidenced in previous studies of patients with TSC,^31^, ^32^ and intellectual disability has been shown to be more common in patients with epilepsy.^32^

All‐cause healthcare resource use was substantial in patients with TSC, with 41% of patients hospitalized at least once in 2018, predominantly to acute care facilities, 66% visiting a general practitioner, and 61% visiting a hospital specialist. These results were in line with previous studies reporting healthcare resource utilization in patients with TSC.^13^, ^17^ In a UK study by Kingswood et al (2016), over a 3‐year period, 73% of patients with TSC attended visits with primary care physicians, and 16% and 93% of patients with TSC had recorded inpatient admissions and outpatient (specialist) visits during this period.^13^ Additionally, a survey by Rentz et al (2016) in the USA reported that in the previous year nearly all patients with TSC (99%) had visited a physician (mean: 22.0 visits), 37% of patients had been hospitalized (2.5 visits), and 28% had visited an emergency room (2.5 visits).^17^

Patients with TSC with epilepsy were found to have greater healthcare resource utilization, particularly hospitalization rates (46% vs 32%) and visits to hospital specialists (65% vs 51%), compared with patients without TSC.^9^, ^10^, ^14^, ^18^ Some previous studies have reported hospitalization rates of around double that in patients with TSC with epilepsy compared with those without epilepsy or comparator cohorts.^9^, ^14^ Strzelczyk et al (2021) reported hospitalization rates of 0.7 vs 0.4 visits per patient year for patients with TSC with and without epilepsy as assessed in Germany over a 10‐year study period.^14^ Similarly, Shepherd et al (2017) reported mean (95% confidence interval) inpatient hospital admissions of 3.4 (2.1, 4.7) vs 1.2 (1.0, 1.4) admissions per patient for patients with TSC with epilepsy compared with a comparator cohort over a 3‐year period in a UK‐based study.^9^ However, a study in Sweden reported healthcare resource use in similar relative proportions to that of this study for patients with TSC with and without epilepsy, with a mean (SD) of 6.2 (4.2) vs 4.7 (4.2) outpatient visits per year and 4.8 (6.3) vs 3.3 (5.6) inpatient days per year for patients with TSC with refractory epilepsy compared with the overall population of patients with TSC.^10^ Furthermore, in a US‐based study, Lennert et al (2013) established that hospitalizations were significantly more frequent in patients with TSC with a neurologic disorder compared with the overall population of patients with TSC (*P* = .0049).^18^

Healthcare resource utilization findings were reflected in substantial annual healthcare costs (€9790 per capita), particularly in patients with epilepsy (€11 413 per capita). Although healthcare costs can vary widely between countries, these findings are in line with previous studies in patients with TSC^15^, ^16^ and TSC with epilepsy.^9^, ^20^ In particular, Shepherd et al (2017) found that patients with TSC with epilepsy incurred annual healthcare costs of £14 335 compared with £4448 in a comparator cohort.^9^ Substantial annual healthcare costs in patients with TSC were estimated in studies based on USA (median, $14 807 across all years studied)^15^ and German (mean, €25 808 annually)^16^ healthcare databases, which identified epilepsy as a predictor of increased total hospital charges (*P* < .001) or cost‐driving factor for total direct costs (*P* = .001)^15^ and nursing care level costs (*P* < .001).^16^ A further retrospective claims‐based analysis in the USA reported higher annual healthcare costs compared with these previously mentioned studies, of between $42 997 and $48 330, with costs of $5335 to $9672 per medically treated seizure event.^20^ Furthermore, a retrospective questionnaire by Grau et al (2021) identified epilepsy as a statistically significant cost‐driving factor for both direct and indirect healthcare costs in children.^33^

In terms of comorbidities, this study reported higher rates of intellectual disability in patients with TSC with epilepsy compared with those without epilepsy (20% vs 5%), which is consistent with previous findings, although the rates of intellectual disability reported here appear to be lower than those of prior studies.^9^, ^11^, ^14^ Strzelczyk et al (2021) reported that patients with TSC with epilepsy had greater incidences of comorbidities than patients without epilepsy, particularly cognitive disabilities (51% vs 8%).^14^ Similarly, an international study by Nabbout et al (2019) reported intellectual disability in 59% of patients with TSC with epilepsy evaluated for intellectual ability,^11^ while Shepherd et al (2017) reported intellectual disability and learning disability in 9% and 54% of patients with TSC with epilepsy.^9^

Many patients with TSC with epilepsy received several different ASMs in 2018, with 74% of patients dispensed ≥1 ASM in 2018, although for these patients it is not known whether ASMs were taken in combination or sequentially within this period. Overall, 27% of patients with TSC with epilepsy had no dispensed ASMs during the last study year, while only 6% of incident patients had no ASMs dispensed during the period from 2006 to 2018. The treatment pattern of ASM dispensations observed here was similar to that observed previously, with the lowest proportions of patients receiving either none or ≥4 ASMs, and the remainder distributed to receive a maximum of between one and three ASMs.^9^, ^12^, ^34^ Some studies did not align to this pattern; for example, Zöllner et al (2021) reported a greater proportion of patients receiving no ASMs (36%),^16^ while Song et al (2018) reported the higher proportion of patients had received ≥5 distinct ASMs (35%)^19^; however, the timeframes over which these data were captured varied greatly (3 months vs 13 years). The use of multiple ASMs observed in the present and previous studies may be attributed to the high proportion of patients with TSC who develop refractory epilepsy.^5^, ^6^, ^9^, ^12^, ^35^ Everolimus is a mammalian target of rapamycin complex 1 (mTORC1) inhibitor, approved internationally as an add‐on treatment for TSC‐associated refractory epilepsy, in addition to previous approval for the treatment of TSC‐associated subependymal giant cell astrocytomas and renal angiomyolipomas, and is understood to have a positive impact on abnormal brain cell growth as well as neural and synaptic activity in patients with TSC.^36^ In this study, everolimus was dispensed to 9% of all patients with TSC yet contributed to 70% of medication costs in patients with TSC with epilepsy. The prescribed medications reported in this study are consistent with currently recommended treatments.^37^, ^38^, ^39^ Similarly, a retrospective questionnaire of direct and indirect costs of TSC in children and adolescents by Grau et al (2021) reported that mammalian target of rapamycin inhibitors, including everolimus, accounted for 46.6% of total direct costs.^33^

Although the mean age at death was lower in patients with epilepsy compared with those without epilepsy in this study, no difference in survival was observed.

### 4.1 Limitations

Limitations of this analysis include uncertainties around patient identification. In particular, some patients with TSC may not have received an LTD registration of Q85.1 (TSC) but may have benefitted from LTD status under the code Q85* (unclassified phakomatoses); such patients would not have been included in the crude prevalence estimation. To address this uncertainty, an upper range estimate including LTD codes of Q85* as probable TSC cases was also reported; however, although exclusions were made, this broader population might include some patients with unspecified neurofibromatosis not associated with TSC.

Since the majority of patients with TSC are followed up in outpatient care, the method of identifying patients with relevant ICD‐10 codes in the inpatient setting could be considered a limitation of this study. However, this identification process was performed over a 13‐year retrospective period, and the impact of TSC suggests that it is unlikely that a patient with TSC had never been admitted in any hospital with ICD‐10 Q85.1 as a primary or secondary diagnosis during this time, even if followed up in outpatient care. Patients without an LTD code may be those who have not yet had a formal diagnosis or who were already benefitting from a full coverage for their comorbidities.

The study may also be lacking data from patients who were living in care facilities or institutions during the study period. Specialized institutions within the French healthcare system may not capture individual patient claims data in the SNDS database if they have an internal pharmacy to deliver medications or if they receive funding on a global budget. Cost and healthcare resource utilization estimates may be biased by the underreporting of data in such patients. The lack of data from such patients may also explain the low rates of intellectual disability in patients with TSC with epilepsy observed in this study, as compared with previous studies.^9^, ^11^, ^14^

The documentation of comorbidities through the identification of LTD diagnosis is perhaps the most important limitation of our study. Conditions eligible for LTD registration belong to a pre‐established list (three‐digit ICD‐10 coded) and each registration must be validated by a medical consultant of the beneficiary's health insurance scheme; therefore, for patients with several concomitant and associated comorbidities, the physician could decide not to declare all as separate conditions. Furthermore, databases such as SNDS have limitations, including the validity of diagnoses and lack of clinical or biological data; however, diagnoses in the SNDS database have, in principle, been approved by physicians, and coding instructions for hospital diagnoses are in place to promote homogeneous data reporting.

### 4.2 Clinical relevance and future directions

The data reported from this retrospective analysis provide valuable insight into the burden of illness for patients with TSC with or without associated epilepsy in the French healthcare system. In terms of future directions, study data suggest that refractory epilepsy causes great burden in patients with TSC with epilepsy, with many patients receiving polytherapy, while healthcare costs and resource utilization remain higher than in patients without epilepsy, thus identifying the need for ASMs that are effective in patients who are resistant to many existing therapies.

### 4.3 Conclusions

TSC with epilepsy was associated with substantial healthcare costs and resource utilization, particularly comprising outpatient and medication costs. Patients with epilepsy had substantially higher annual healthcare costs (both inpatient and outpatient costs) compared with patients without epilepsy, including higher medication costs due to everolimus uptake. The number of patients dispensed multiple ASMs suggested a high rate of refractory epilepsy in this population. Although this analysis is limited by uncertainties around patient identification and use of LTD registration to document comorbidities, this is the largest retrospective population‐based study of TSC in a French population, reporting prevalence and incidence rates of TSC that are consistent with that of previous literature.

## CONFLICT OF INTEREST

FF, CL, and MZ are employees of Cemka. JM is an employee of Jazz Pharmaceuticals, Inc.

## ETHICS STATEMENT

We confirm that we have read the Journal's position on issues involved in ethical publication and affirm that this report is consistent with those guidelines.

## Supporting information


Appendix S1
