# Supplementary material for: Epidemiology and disease burden of tuberous sclerosis complex in France: A population‐based study based on national health insurance data
Source: Epilepsia Open. 2022 Aug 27;7(4):633–44. doi: 10.1002/epi4.12636 (PMC9712482; doi:10.1002/epi4.12636)
Supplement: Supplementary file 1 — Appendix S1 [file EPI4-7-633-s001.docx]

**Supplementary Materials for**

**Epidemiology and disease burden of tuberous sclerosis complex in France: a population-based study based on national health insurance data**

Francis Fagnani^1^, Caroline Laurendeau^1^, Marie de Zelicourt^1^, Jade Marshall^2^

^1^Cemka, Bourg-la-Reine, France; ^2^Jazz Pharmaceuticals, Inc., London, UK

**Corresponding author**: Francis Fagnani, Cemka, 43 Boulevard du Maréchal Joffre, 92340 Bourg-la-Reine, France, [Francis.Fagnani@cemka.fr](mailto:Francis.Fagnani@cemka.fr)

**FIGURE S1** Age distribution of patients with probable TSC in 2018 (prevalent population, all patients)


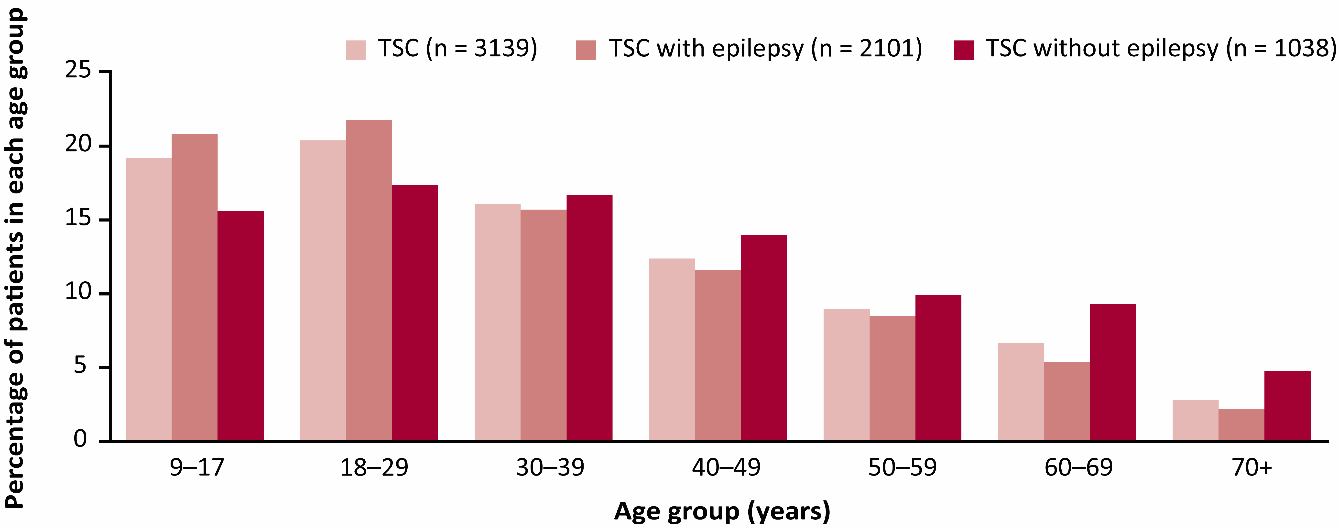


TSC, tuberous sclerosis complex

**TABLE S1** Annual healthcare costs for patients with TSC in 2018 (prevalent population, patients with recorded costs)

|  | **TSC (N = 2936)** | | **TSC with epilepsy (n = 1989)** | | **TSC without epilepsy (n = 947)** | |
| --- | --- | --- | --- | --- | --- | --- |
|  | **Mean (SD) per capita annual cost (€)** | **% of total costs** | **Mean (SD) per capita annual cost (€)** | **% of total costs** | **Mean (SD) per capita annual cost (€)** | **% of total costs** |
| **Total costs** | **9790 (24531)** | **100** | **11413 (27620)** | **100** | **6382 (15706)** | **100** |
| **Inpatient care** | **3710 (19311)** | **38** | **4208 (22234)** | **37** | **2665 (10791)** | **42** |
| Acute care facilities | 2205 (6752) | 59* | 2346 (6289) | 56* | 1910 (7627) | 72* |
| Psychiatry | 794 (7506) | 21* | 886 (7633) | 21* | 600 (7231) | 23* |
| Rehabilitation | 701 (15857) | 19* | 970 (19191) | 23* | 137 (2399) | 5* |
| **Outpatient care** | **6080 (12868)** | **62** | **7205 (14227)** | **63** | **3717 (8954)** | **58** |
| Medication | 3575 (10589) | 59^†^ | 4518 (12102) | 63^†^ | 1595 (5861) | 43^†^ |
| *ASMs* | *352 (875)* | 10^‡^ | *519 (1021)* | 12^‡^ |  |  |
| *Everolimus* | *2414 (10092)* | 68^‡^ | *3171 (11614)* | 70^‡^ | *823 (5370)* | 52^‡^ |
| Physician visits^§^ | 699 (3298) | 12^†^ | 640 (2866) | 9^†^ | 823 (4058) | 22^†^ |
| Transportation | 627 (2824) | 10^†^ | 710 (2890) | 10^†^ | 453 (2673) | 12^†^ |
| Medical devices | 393 (1358) | 7^†^ | 444 (1511) | 6^†^ | 287 (949) | 8^†^ |
| Other HCPs | 331 (1171) | 5^†^ | 392 (1310) | 5^†^ | 203 (791) | 6^†^ |
| Laboratory test | 136 (303) | 2^†^ | 130 (299) | 2^†^ | 146 (312) | 4^†^ |
| Dental care | 74 (305) | 1^†^ | 69 (320) | 1^†^ | 85 (271) | 2^†^ |
| Other | 245 (3162) | 4^†^ | 302 (3527) | 4^†^ | 125 (2203) | 3^†^ |

*percentage of inpatient care costs; ^†^percentage of outpatient care costs; ^‡^percentage of medication costs; ^§^including hospital

ASM, antiseizure medication; HCP, healthcare professional; TSC, tuberous sclerosis complex, SD, standard deviation
